# Effects of heat-moisture extrusion on the structure and functional properties of protein-fortified whole potato flour

**DOI:** 10.1016/j.fochx.2024.102048

**Published:** 2024-11-28

**Authors:** Suying Hao, Ying Zheng, Mingyuan Li, Xiaobo Feng, Xiaoqing Yang

**Affiliations:** College of Food Science and Engineering, Inner Mongolia Agricultural University, Hohhot, Inner Mongolia 010018, China

**Keywords:** Heat-moisture extrusion, Whole potato flour, Exogenous protein, Physical modification, Functional properties

## Abstract

The aim of this study was to explore the effects of twin-screw extrusion combined with exogenous protein on interaction between starch and protein of the compound potato powder thereby contributing to better structural characterization and functional properties of whole potato flour lack of gluten. The results showed that the heat-moisture extrusion increased the relative crystallinity of starch. Meanwhile, the exogenous protein introduced (-SH_free_) under the action of extrusion to form (-S-S-) in the compound system and furtherly promoted the construction of the network structure of the protein. Additionally, WAC, OAC, solubility, the thermal properties and the thermal stability of the compound potato powder were improved. The content of bound water was increased, thereby increasing water migration in the dough. The results confirmed effectiveness of using extrusion combining with exogenous protein to improve the physicochemical properties of potato flour.

## Highlights

• The extrusion benefits constructing network structure in starch-protein compound system.
• The physicochemical properties of gluten-free potato powder were ameliorated.
• The modification of starch and protein affects the thermal stability of the powder.
• High temperature has great influence on the properties of compound powder.

## 1 Introduction

Whole potato flour is obtained by processing potato tubers through multiple steps followed by dehydration drying. It covers all the dry matter of fresh potato tubers except potato peel, and the aroma and nutrients of potatoes are also preserved completely(Hou et al., 2023). Potato flour mainly consists of starch, the content of which is more than 60 %. Potato flour is rich in vitamin C, potassium, and polyphenols(Song et al., 2023) and does not contain cholesterol. Whole potato flour can be used directly as the final product and can also be used as a basic raw material to make various deep-processed foods(Mu & Sun, 2017). Gluten protein is absent in whole potato flour, making it an ideal choice for creating gluten-free products. It can be used to make functional foods suitable for people with gluten allergy. However, as it lacks gluten, constructing network structure in the potato flour dough is difficult(Yang et al., 2023). The absence of suitable network structure results in poor thermal stability and water-holding capacity, and difficult product molding and processing(Yin et al., 2021). Whole potato flour has poor processing performance, which hinders its development and application in food. Nowadays, the main method of whole potato flour food processing involves mixing wheat flour rich in gluten and gliadin with whole potato flour to compensate for the processing defects of insufficient gluten protein in potato flour (Sun et al., 2020). Gluten, composed of gliadin and glutenin, is involved in the formation of a gluten network and improves the viscoelasticity of the dough (Barak et al., 2013), but it is unsuitable for people with gluten allergy. Potato protein (Fu et al., 2020) and rice protein (Shi et al., 2024) are high-quality plant proteins with a high nutritional value, suitable amino acid composition balance, high amino acid content, low allergic effects, anti-oxidant, anti-hypertensive properties and other characteristics. The two kinds of protein can improve the solubility, water-holding capacity, oil-holding capacity, emulsifying properties and foaming stability of products (Zheng et al., 2024). In addition, potato protein homologous to potato flour can be selected to have better matching compatibility with gluten-free base material. Previous studies have confirmed that the addition of potato protein and rice protein as exogenous proteins has a good effect on optimizing the dough functional properties, like the functional role of gluten(Xu et al., 2022). This study intends to explore whether these two proteins can transform their own sulfhydryl groups into disulfide bonds by means of modification methods, thereby constructing gluten network akin to wheat dough and further replace the function of gluten protein.

The twin-screw extrusion process is a physical modification method that applies a strong mechanical force and forces materials to pass through the screw and formed by the mold under high temperature and pressure. During extrusion, the structure (Cai et al., 2022) and properties (Hossain et al., 2021) of the macromolecular substances, such as starch and protein, can be changed due to the action of pressure, temperature and shear force. The protein will undergo structural cleavage and unfolding. The amylopectin in the starch will be rapidly cleaved, the crystallinity will be reduced, and the proportion of amylose will be increased, thereby changing the original binding state of the protein and starch and improving the processing performance. Cheng (Cheng et al., 2023) found that extrusion treatment can increase the gelatinization degree, water absorption index and water solubility index of starch and also improve the performance of dough. The above-mentioned studies showed the alteration of pure starch or protein because of extrusion treatment. However, starch and protein coexist during food processing. When starch is processed, it is converted from a natural particle state to a gelatinization state. Protein denaturation and the rupture of starch particles during extrusion could result in additional interactions between two types of polymers. The interaction of starch and protein in food occurs via covalent bonds, electrostatic interactions, hydrogen bonds, hydrophobic interactions(Yu et al., 2023). Under the physical modification effect of extrusion, not only do the physical and chemical properties of starch and protein change significantly, but the crosslinking polymerization between them also becomes strong or weak, which in turn affects the processing characteristics and product quality of food. However, only a few studies have investigated this aspect.

This paper acims to study the effect of twin-screw extrusion treatment along with the addition of exogenous proteins on constructing gluten network akin to wheat dough and furtherly approaching the function of gluten protein in wheat dough, thereby to improve the overall processing performance of the compound potato powder by analyzing their structure, physicochemical properties, thermal properties, thermal degradation and water migration. The study intends to promote the development of potato whole flour gluten-free functional food.

## 2 Materials and methods

### 2.1 Materials

Potatoes (*Solanum tuberosum* L. *cv. Shepody*) were purchased from Wuchuan County Potato Planting Base in (Hohhot,China) and processed to form native whole potato powder by Heilongjiang Beidahuang Potato Industry Co. (Harbin, China), which contained 12 %(db) ± 0.05 crude protein, 0.02 %(db) ± 0.01 fat, 26 %(db) ± 0.11 carbohydrate and 12.1 % ± 0.01 moisture. Potato protein powder was analytical grade, which protein content was 90 %. Rice protein powder was analytical grade, which protein content was 90 %. The two protein powders were purchased from Aladdin Co. Ltd., Shanghai, China. All chemicals were analytical grade and purchased from Xi'an Michelle Biotechnology Co., Ltd., China.

### 2.2 Extrusion modification treatment of compound potato powder

First, the exogenous protein of 2 % potato protein powder and 3 % rice protein powder were simultaneously added to the potato whole flour to form a compound powder and deionized water was used to make a water content of 30 % after the twin-screw extrusion treatment (DSE32-experimental twin-screw extrusion extruder with motor power 5.5 KW, electric heating style, Φ32 screw diameter and 2:3 of screw length-diameter ratio. Jinan Datong Machinery Equipment Co., Ltd.), which was based on the screening pre-experiments of water content. The extrusion parameters were set as follows: screw speed 120, 180 and 240 r/min (denoted as S1-PF, S2-PF and S3-PF), extrusion temperature 80, 100 and 120 °C (denoted as T1-PF, T2-PF and T3-PF). The blank group (denoted as P) included native potato flour without any addition nor extrusion treatment and the control group (denoted as PF) was the potato flour without extrusion treatment but only with added potato protein and rice protein powder. A certain amount of samples from the blank group, control group and each group of extrudes were respectively weighed and freeze-dried for 24 h. The freeze-dried samples were crushed and pulverized by 100 mesh sieve and stored at 4 °C for further measurement.

### 2.3 Content of free sulfhydryl group (-SH) and disulfide bond (-S-S-)

The contents of free sulfhydryl groups (-SH) and disulfide bonds (-S-S-) in the samples were slightly modified according to the method of Gao et al.(Gao et al., 2021) The freeze-dried sample was 150 mg, guanidine hydrochloride 4.7 g and 0.2 M of TRIS-GLY (pH 8.0, 6.9 g glycine, 1.2 g EDTA, 10.4 g Tris per liter) buffer was used to volume to 10 ml.

To determine the content of free sulfhydryl (-SH_free_), mixing 1 ml of the sample solution with 4 ml of solution A (containing 8 M of urea and 5 M of guanidine hydrochloride), mix well and add 0.1 ml of Ellman's solution (containing 4 mg/l of 5,5 ‘-dithiobis [2-nitrobenzoic acid] (DTNB)). The absorbance was measured by ultraviolet spectrophotometer (U-2910, Hitachi, Japan) at 412 nm after the reaction at room temperature for 30 min.

The total sulfhydryl (-SH_total_) content was determined by adding 4 ml solution A and 0.1 ml β-mercaptoethanol to 1 ml sample solution. After the mixture had been maintained at room temperature for 1 h, 12 % trichloroacetic acid was introduced to prolong the reaction for another hour. The precipitation was washed twice with trichloroacetic acid and centrifuged at a speed of 5000 r/min for a duration of 10 min following each wash. Dissolve the precipitate in 8 M urea, add 0.08 ml Ellmen's, take out 1 ml, add 4 ml Tri-Gly buffer, mix well and measure absorbance at 412 nm. The values of (-SH) and (-S-S-) are determined using the following formula.

(1) $$-{\mathrm{SH}}_{\text{total}\left(\text{free}\right)}=73.53A_{412}\frac{D}{C},\text{μmol}/g$$

(2) $$-S-S-=\frac{\left(-{\mathrm{SH}}_{\text{total}}\right)-\left(-{\mathrm{SH}}_{\text{free}}\right)}{2},\text{μmol}/g$$

here, A_412_ is the absorbance at 412 nm; D is dilution factor; C is the sample concentration (mg/ml).

### 2.4 X-ray diffractogram (XRD)

The changes in the crystal structure and relative crystallinity of freeze-dried sample powder were analyzed using a multifunctional X-ray diffractometer (D8 ADVANCE Da Vinci, Bruker, Germany), following the procedures reported by Shi(Shi et al., 2018). The test conditions were as follows: scanning at 2θ angle of 5–35°, emission voltage of 40 kV, emission current of 40 mA and scanning rate of 20°/min.

### 2.5 Fourier transform infrared spectroscopy (FT-IR)

The infrared spectra of freeze-dried sample powders were determined using an infrared spectrometer (IR Tracer 100, Shimadzu, Japan). The scanning range was set to 400–4000 cm^−1^, the resolution was 4 cm^−1^ and the number of scans was 32.

### 2.6 Scanning electron microscopy (SEM)

Scanning electron microscopy (SEM, TM4000Plus, Hitachi, Japan) was used to examine the surface morphology of the samples. The cross-section of the uncrushed sample after freeze-drying was sprayed with gold and the sample was observed at 500 × magnification.

### 2.7 Confocal laser scanning microscopy (CLSM)

The microstructure of the sample was observed by confocal laser scanning microscopy (TCS SP8 STED 3×, Leica, Germany), following the method described by Lucas (Lucas et al., 2018). First, 50 g of freeze-dried sample powder was taken from each group. Then, the sample and water were mixed in a 2:1 ratio for 10 min to obtain a smooth and uniform dough. The fresh dough was cut into 1 cm^3^ cubes and stored at −18 °C for 24 h after conducting a sampling test. The dough was cut into 1-mm-thick slices, and the starch and protein in the samples were stained with 0.25 % fluorescein isothiocyanate (FITC) and 0.025 % rhodamine B, respectively. The excitation wavelength of FITC was 488 nm and the emission wavelength was 518 nm. The excitation wavelength of Rhodamine B was 568 nm and the emission wavelength was 625 nm.

### 2.8 Water absorption capacity and oil absorption capacity

The water absorption capacity (WAC) and oil absorption capacity (OAC) of the sample were determined following the method described by Xie (Xie et al., 2019), with slight modifications. Briefly, 1 g of freeze-dried sample powder from each group was placed in two centrifuge tubes, to which 10 ml of distilled water and 10 ml of soybean oil were added and the mixtures were stirred for 30 min. Then, they were centrifuged at 4000 r/min for 25 min and weighed after discarding the supernatant. The water absorption index and the oil absorption index were respectively calculated by the following equations:

(3) $$\mathrm{WAC}=\frac{m_{1}-m_{2}}{m_{0}}$$

(4) $$\mathrm{OAC}=\frac{m_{1}-m_{2}}{m_{0}}$$

here, m_0_ indicates dry starch mass (g), m_1_ indicates the mass of the sample and centrifuge tube before centrifugation (g), and m_2_ indicates the mass of the precipitate and centrifuge tube after centrifugation (g).

### 2.9 Solubility and swelling capacity

The solubility and swelling capacity were determined by referring to the method by Wang(M. T. Wang, Liu, et al., 2023). Each group of freeze-dried sample powder (dry weight: 2 g) was dispersed in distilled water to produce a 2 % (*w*/*v*) starch suspension. The suspension was heated in a water bath at 95 °C for 30 min under continuous stirring and cooled to room temperature, followed by centrifugation at 5000 r/min for 20 min. The supernatant was separated and evaporated at 105 °C to obtain the precipitate. The solubility (S) and swelling capacity (SC) were determined using eqs. (3), (4).

(5) $$S=\frac{W2}{W}\times 100\%$$

(6) $$\mathrm{SC}\left(g/g\right)=\frac{W1}{W\times \left(100-S\right)}$$

here, W indicates the dry weight of the sample (g), W_1_ indicates the weight of the precipitate after evaporation (g) and W_2_ indicates the weight of the supernatant (g).

### 2.10 Thermal properties

A differential scanning calorimeter (DSC 25, TA Instruments, USA) was used to analyzed the thermal properties based on the method described by Liu(Liu et al., 2022). To each group of freeze-dried sample powder (2 mg), distilled water (6 μL), and the mixture was added in sealed crucible (TA Q20 liquid Φ 5.4*2.6 mm) at room temperature and incubated overnight. An empty crucible was used as the control. The temperature was increased from 25 to 150 °C and the heating rate was 10 °C/min.

### 2.11 Thermogravimetric test

A thermogravimetric analyzer (TGA8000, PerkinElmer, USA) was used for obtaining TG and DTG curves to assess thermal degradation of samples during thermal procedure. The residual mass fraction of the samples was determined from the TG curve and the mass loss rate was analyzed using the second derivative thermogravimetry (DTG) analysis. About 2.00 mg of each group of freeze-dried sample powder was placed in a ceramic crucible for TGA, then the sample was placed in the automatic loading plate of TGA. The flow rate of nitrogen was established at 10 ml/min, the heating rate was 10 °C/min and the heating range was 20–600 °C.

### 2.12 Low-field nuclear magnetic resonance (LF-NMR)

The water distribution of the samples was analyzed using a low-field nuclear magnetic resonance imaging analyzer (MesoMR23-060H-I, Niumag, Suzhou) (Xie et al., 2023). Briefly, 1 g of fresh dough prepared in Section 2.7, the transverse relaxation time (T_2_) was measured by CPMG pulse sequence. The parameters were as follows: the echo time (TE) was 0.500 ms, the number of sampling points (TD) was 44,030 and the cumulative number (NS) was 32.

### 2.13 Statistical analysis

All experiments were repeated at least thrice. The original data and drawings were analyzed using the Origin software (OriginLab, US, version 2021). Group variances were identified through a one-way analysis of variance using the SPSS 26 software (IBM, Chicago, IL, USA). All differences were considered to be statistically significant at *p* < 0.05.

## 3 Results and analysis

### 3.1 Content of free sulfhydryl group (-SH_free_) and disulfide bond (-S-S-)

**Fig. 1:**
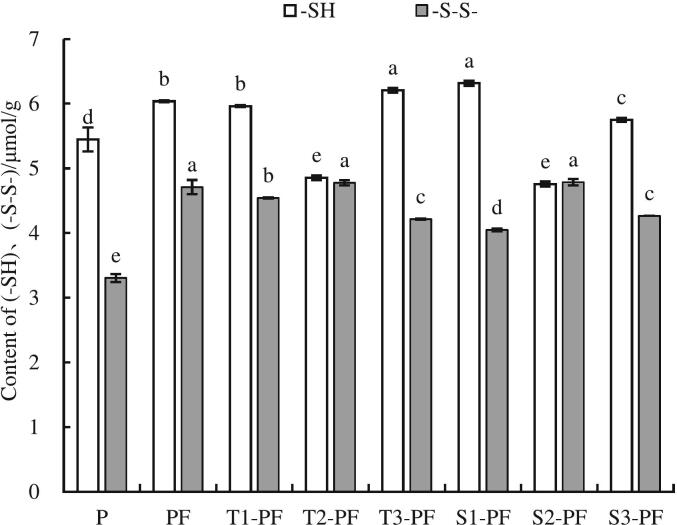
The content of (-SH)、(-S-S-) of protein-fortified potato flour extruded at different temperatures and screw speeds. Note: All experiments were repeated at least thrice. The data are expressed as mean ± standard deviation, and different letters within a column are significant differences (*p* < 0.05).

The contents of (-SH_free_) and (-S-S-) are shown in Fig. 1. the addition of protein in PF increased the content of (-SH_free_) compared with P, indicating that the addition of protein introduced additional sulfhydryl groups and also increased the possibility of disulfide bond formation. The content of (-S-S-) in PF and extruded samples was significantly increased compared with P (*p* < 0.05). Repeated folding and pressing during dough treatment make (-SH_free_) contact to form (-S-S-), which in turn produce network structure that can enhance the dough structure. During extrusion, the intense shear force disrupts starch granules, loosen the structure of both starch and protein, cause the natural curly structure of protein to unfold, boosts the count of (-S-S-) bonds stabilizing its spatial arrangement, reduces the count of free thiol groups (-SH_free_) and facilitates the formation of larger molecular weight protein aggregates. (Yin et al., 2023). In addition, squeezing and shearing exposes proteins to more sulfhydryl groups, which oxidize into new disulfide bonds under the action of O_2_. The content of (-S-S-) in T2-PF and S2-PF samples reached the highest level, but the content of (-S-S-)did not increase continuously. The content of (-S-S-) in T3-PF and S3-PF samples decreased. The possible reason was that excessive extrusion at high temperature and higher screw speed destroyed the starch too much, and the protein aggregated, which made the originally exposed sulfyl groups re-wrapped. In addition, extrusion shearing will also cut the (-S-S-) between protein molecules, thus reducing the number of (-S-S-).

### 3.2 X-ray diffractogram (XRD)

**Fig. 2:**
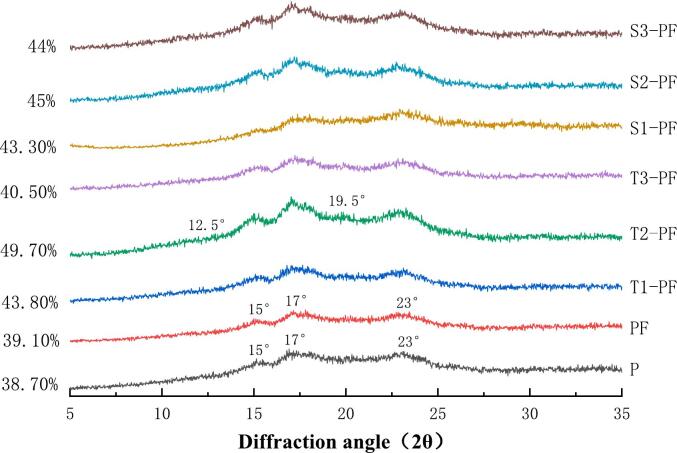
The XRD pattern of protein-fortified potato flour extruded at different temperatures and screw speeds.

The X-ray diffraction patterns of the extruded samples at different temperatures and screw speeds and the relative crystallinity of starch are presented in Fig. 2. According to the XRD pattern, the diffraction peaks of P and PF were highly consistent, which indicated that adding exogenous protein changes neither the peak intensity nor crystal form of the raw whole potato flour. Strong diffraction peaks were recorded at 15.5°, 17.5° and 23.1°, and weak diffraction peaks were recorded at 5.3° and 16°, which were the characteristic peak of B-type starch(Chen et al., 2019). The X-ray diffraction patterns of T1-PF, T2-PF, T3-PF, S1-PF, S2-PF, and S3-PF in the test group showed that the characteristic peaks were enhanced or weakened, indicating that potato starch was gelatinized and protein was denatured, but the position of the diffraction peak did not change significantly. When 2θ was 12.5° and 19.5°, V-shaped characteristic peaks appeared in the extruded compound potato powder. The V-shaped structure was related to the formation of complexes between gelatinized amylose and lipids. The crystalline structure of potato starch was destroyed due to the high temperature and high pressure in the extruder (starch gelatinization), but within a certain range, as the temperature and screw speed increased, the characteristic peaks at 2θ of 12.5° and 19.5° were enhanced. A prominent characteristic protein peak of 19.5° was detected in the XRD spectrum of the compound powder and the intensity of the starch absorption peak (17° and 23°) decreased, which occurred probably because of the interaction between starch and more proteins. The appearance of the protein absorption peak indicated that the two biological macromolecules were biocompatible (Escamilla-García et al., 2013). The relative crystallinity of T2-PF increased to 49.70 % compared to the relative crystallinity of P (38.70 %). This finding showed that more protein-starch interactions may lead to crosslinking, ordered rearrangement between molecules and tight binding through covalent/non-covalent bonds to produce more new protein-starch complexes, comparable outcomes were reported by Kristiawan (Kristiawan et al., 2018). The screw extrusion treatment first led to the destruction of the amorphous region with weak starch structural force through the combined action of pressure, temperature and shear force. With the rise in extrusion temperature and screw speed, there was initially an increase in relative crystallinity, followed by a decrease.(Fig. 2). High temperature and higher screw speed destroyed the crystalline inner layer of starch and the double helix structure of the crystalline region was partially destroyed, resulting in a decrease in relative crystallinity (Fig. 2:T3-PF-40.5 %, S3-PF-44 %). Additionally, high shear force and high heat destroy the original hydrogen bond between starch particles, resulting in the unwinding of the double helix structure, thus reducing the ordered structure and changing the original morphological stability of starch (Wang, Wang, et al., 2023). Under appropriate conditions, the relative crystallinity increased with the increase in temperature and screw speed, which might be due to the rearrangement of starch molecules caused by screw extrusion treatment and the orderliness of internal structure increased slightly(Zhang et al., 2018), delaying starch retrogradation.

### 3.3 FT-IR spectroscopy

**Fig. 3:**
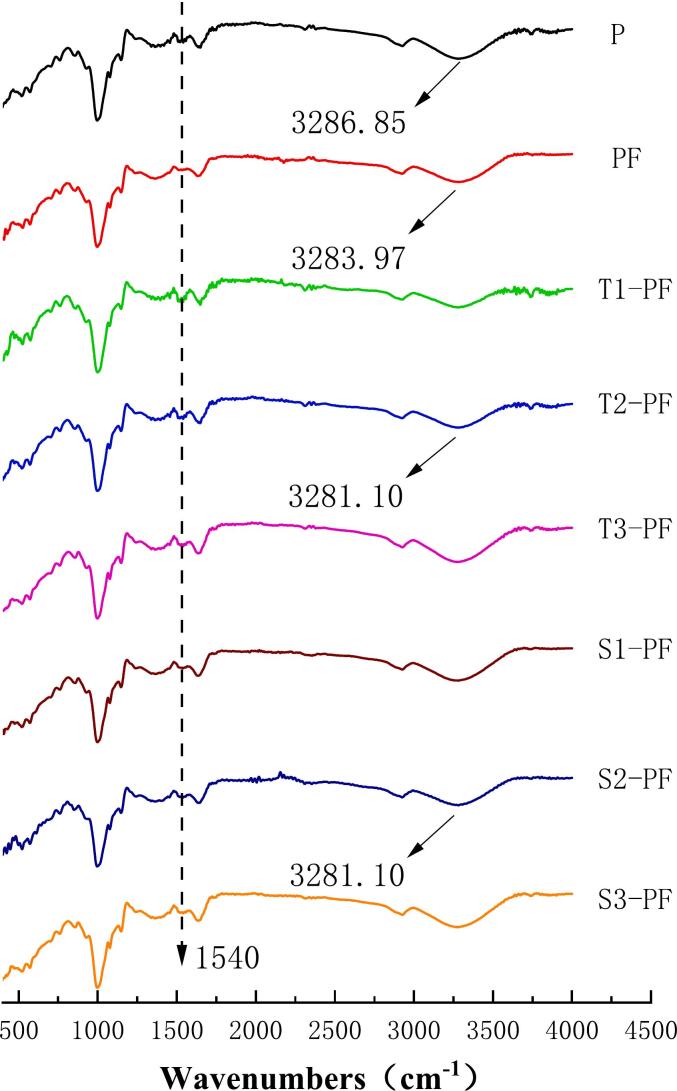
The FT-IR pattern of protein-fortified potato flour extruded at different temperatures and screw speeds.

The position of the peak and the intensity of the change in the infrared spectrum are determined by the chemical environment and the form of vibration of the chemical bond in the internal molecular chemical bond of the measured substance. The infrared spectra of the compound potato powder after extrusion treatment at different temperatures and screw speeds are shown in Fig. 3. No significant change was found in the characteristic peaks of T1-PF, T2-PF, T3-PF, S1-PF, S2-PF and S3-PF in the experimental group compared to the corresponding peaks in the P and PF groups. This suggests the absence of any new group formation in the compound system after extrusion treatment and no significant change occurred in the molecular structure; this confirmed that the screw extrusion treatment was a kind of physical modification. The peak position of the unextruded mixture is almost the same as that of the extruded compound powder, which indicates that the hydroxyl group in the starch only reacts with the specific amino acids in the protein. The absorption peak intensity of the spectra of T1-PF, T2-PF, T3-PF, S1-PF, S2-PF and S3-PF were higher than those of P and PF about 1540 cm^−1^, which was attributed to the Maillard reaction between small molecular sugars produced by starch degradation during extrusion and protein (Guerrero et al., 2011). Its strength decreased as the temperature increased, indicating that higher temperatures affected the interaction of the starch-protein mixed system. The screw speed had a negligible effect on it and the peak value remained at the same level. During extrusion, the interaction between protein and starch led to a shift of the characteristic peaks.

### 3.4 Morphological properties

**Fig. 4:**
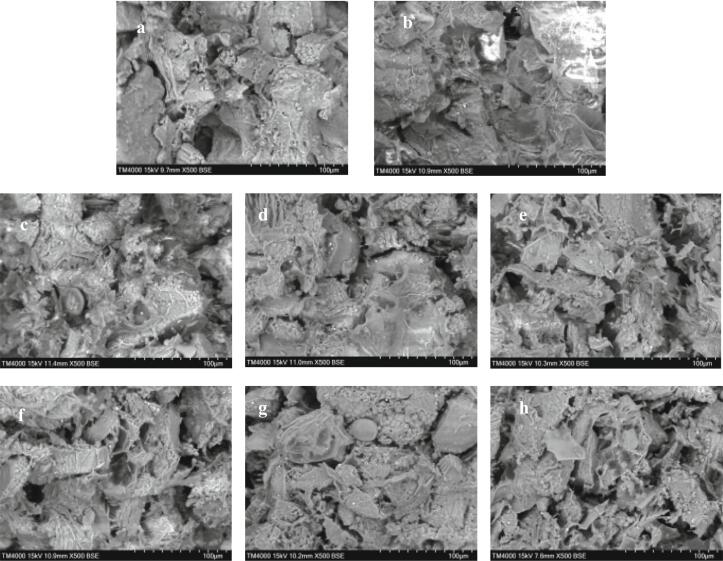
SEM images of protein-fortified potato flour extruded at different temperatures and screw speeds. (a-P; b-PF; c to e - T1-PF, T2-PF, and T3-PF; f to h - S1-PF, S2-PF, and S3-PF)

The surface morphology of the sample was directly examined via SEM. The effects of extrusion temperature and screw speed on the morphology of compound powder particles were studied by observing the microstructure of compound potato powder after heat-moisture extrusion treatment. The structure of P (Fig. 4a) was loose, and cracks and holes were common. In PF (Fig. 4b), adding exogenous protein improved the dough structure, the dough became smoother and the holes became smaller. After extrusion treatment, the protein formed a light and thin filamentous protein network structure. These filamentous protein network structures connect each starch block or wrap the starch granules. At the same screw speed, more dispersed and smooth filamentous protein networks were observed in T2-PF (Fig. 4d). During extrusion, the structure of the protein was stretched, the size of the starch granules decreased significantly, and the small granules of starch were dispersed irregularly inside the dough, which decreased the cracks in the dough. The protein interacted with the starch, showing a mixed block and granular state. The protein network covered the surface of the starch and connected the cracks in the dough. However, the structure of starch and protein in T3-PF deteriorated, and the granules were severely damaged and degraded at high temperature. The small starch granules aggregated into blocks and the surface lost its smoothness. The cleavage of -S-S- decreased the strength of the network structure (Fig. 4e), which matched the findings of Ali (Ali et al., 2020) who reported that the microstructure of starch extruded at 100 °C was denser than that extruded at 130 °C and 160 °C. The results are consistent with the reduction of T3-PF -S-S- number in 3.1. At the same temperature, as the screw speed increased, the dough showed a prominent filamentous protein network that encapsulated the starch granules (Fig. 4f-h). When the screw speed was very high, the structure becomes coarse and the network structure becomes relatively poor, which may be because the protein and starch are damaged after extrusion, causing partial starch aggregation and weak interaction between protein and starch. (Fig. 4h). The protein network structure in S2-PF is smoother and more uniform (Fig. 4g).

### 3.5 Confocal laser scanning microscopy (CLSM)

**Fig. 5:**
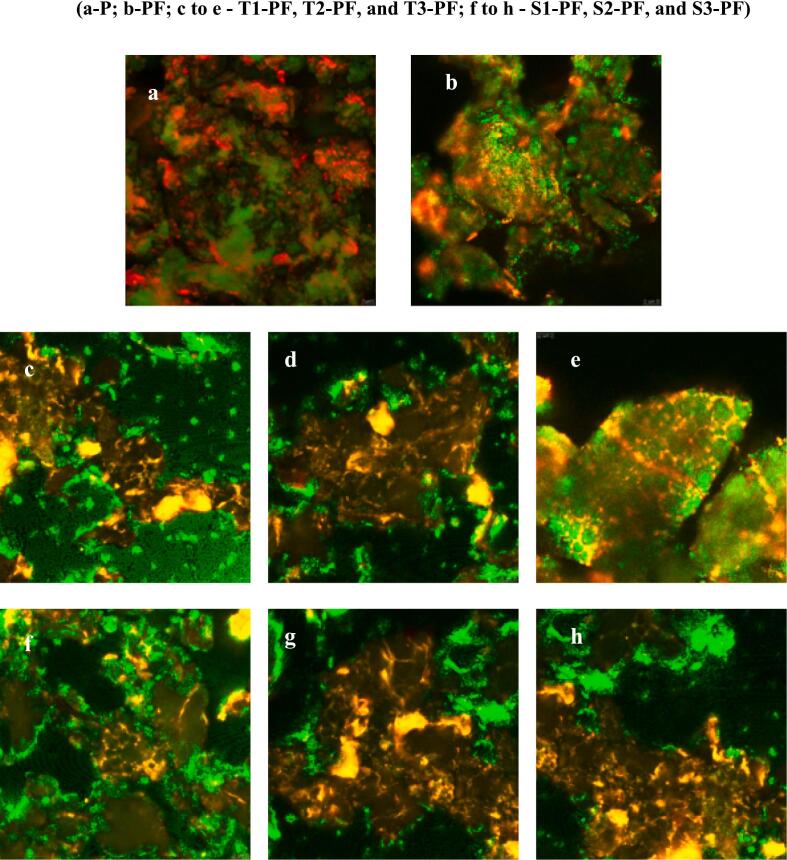
CLSM diagram of protein-fortified potato flour extruded at different temperature and screw speeds. Where, the a is P, b is PF, c to e is respectively T1-PF, T2-PF and T3-PF, and f to h is respectively S1-PF, S2-PFand S3-PF. Double staining with Rhodamine B and FITC was used to distinguish gluten protein (yellow strands) and starch granules (in green). (For interpretation of the references to colour in this figure legend, the reader is referred to the web version of this article.)

The network structure of the sample was visualized by laser confocal microscopy (Fig. 5). In P (Fig. 5a), the starch granules were more dispersed, not broken and experienced no mechanical damage. PF showed the effect of adding proteins (Fig. 5b). The starch particles appeared smaller with edge morphology. After mixing evenly with proteins, starch particles and proteins were stacked, making the dough structure tight. After extrusion treatment, the particles of T1-PF, T2-PF, T3-PF, S1-PF, S2-PF and S3-PF changed significantly, and the particle diameter decreased significantly. The starch system captured and bound the protein, making the complex structure more uniform and compact (Fig. 5c-h). The protein aggregated to form the network structure. These networks were attached to the surface of the starch or connected to it. The protein caused the starch particles to disperse and hindered the interaction between the starch molecules. When the temperature and screw speed were within a certain range, the proteins in T1-PF, T2-PF, S1-PF and S2-PF were uniformly wrapped with starch to form a dense network and connected starch granules. As the temperature and screw speed increased, the protein network density increased. During aggregation, the modified protein was stabilized by hydrophobic interactions and (-S-S-)(Mann et al., 2014). When the temperature and rotation speed reached the maximum value, T3-PF (Fig. 5e) and S3-PF (Fig. 5h) showed that the temperature strongly influenced structural damage and the protein aggregation was distributed unevenly in the whole structure of the dough. Although the screw speed was very high, the network structure was destroyed by extrusion, but a few network structures were still retained. Additionally, extrusion treatment smashed the starch granules and decreased their size. Moreover, the starch granules were irregularly dispersed in the dough. Therefore, partial starch aggregation also occurred, which matched the results of SEM.

### 3.6 Water absorption and oil absorption capacity

**Fig. 6:**
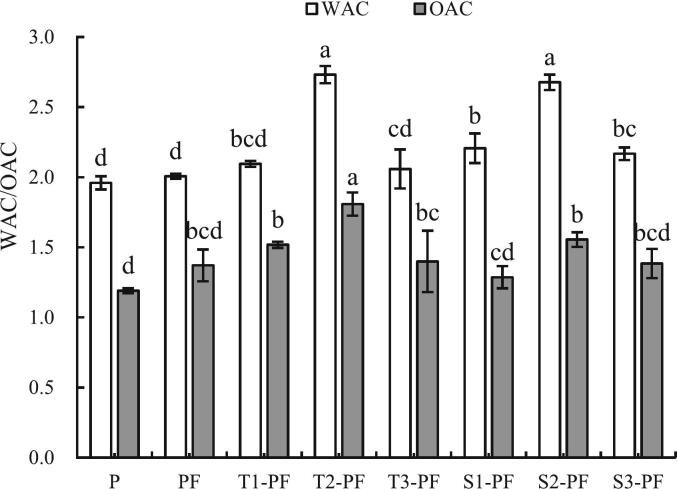
Water absorption and oil absorption capacity of protein-fortified potato flour extruded at different temperatures and screw speeds. Note: All experiments were repeated at least thrice. The data are expressed as mean ± standard deviation, and different letters within a column are significant differences (*p* < 0.05).

The effect of extrusion treatment on the WAC and OAC of protein-fortified potato flour is shown in Fig. 6. The water absorption index of starch reflects its ability to bind to water, which indirectly indicates the degree of non-denaturation of starch structure or the degree of starch gelatinization(Oh et al., 2018). The WAC of PF was higher than P, although the differences was not significant (*p*>0.05). The WAC of the samples after extrusion treatment changed to different degrees, among which the WAC of T2-PF and S2-PF increased significantly (∼39.39 %; *p* < 0.05). The protein was exposed to the dual effects of heat and machinery in the extruder, causing partial aggregation and expansion of the protein molecules, destruction of the spatial structure, weakening of the hydrophobic interaction between the protein macromolecules and an increase in the WAC. Extrusion treatment destroyed the starch granules, exposing more hydrophilic hydroxyl groups; additionally, the hydrogen bonds formed by water molecules increased, which led to an increase in the WAC after extrusion. Additionally, the surface of the granules became rough, the specific surface area increased and the ability to adsorb water increased. As the temperature and screw speed increased, the WAC of T1-PF, T2-PF, T3-PF, S1-PF, S2-PF, and S3-PF in the experimental group first increased and then decreased. These changes occurred because, during extrusion, the shear force caused the natural structure of the protein to expand, the number of disulfide bonds that maintained its spatial conformation increased, the number of sulfhydryl groups decreased(Tang, 2019), the (-SH_free_) in the protein were promoted to transform into disulfide bonds, and a network structure was formed, which bound more water(Xu et al., 2022). However, extrusion at a very high temperature and screw speed caused the protein to aggregate, destroyed the integrity of the formed network structure and decreased the ability to bind water. Following the destruction of the protein structure through extrusion treatment, the hydrophobicity of the protein increased, which partly affected the WAC of the compound powder.

The OAC reflects the emulsifying ability of starch. The OAC of PF was significantly higher than P and the exogenous protein improved the OAC of the composite system. The OAC of the samples after extrusion treatment changed to different degrees, and the OAC of T2-PF was 51.19 % higher than that of P. Similar to the WAC, the main reason for the improvement in the OAC was that the starch granules were crushed by extrusion, which resulted in protein denaturation and the exposure of more binding sites. With the increase in the extrusion temperature and screw speed, the OAC first increased and then decreased, and the temperature significantly affected the capacity. Starch agglomerates at higher temperatures. In our experiment, high extrusion temperatures caused crystal rearrangement of broken starch, covering the binding sites of oil molecules and affecting the OAC of starch.

### 3.7 Solubility and swelling capacity

**Fig. 7:**
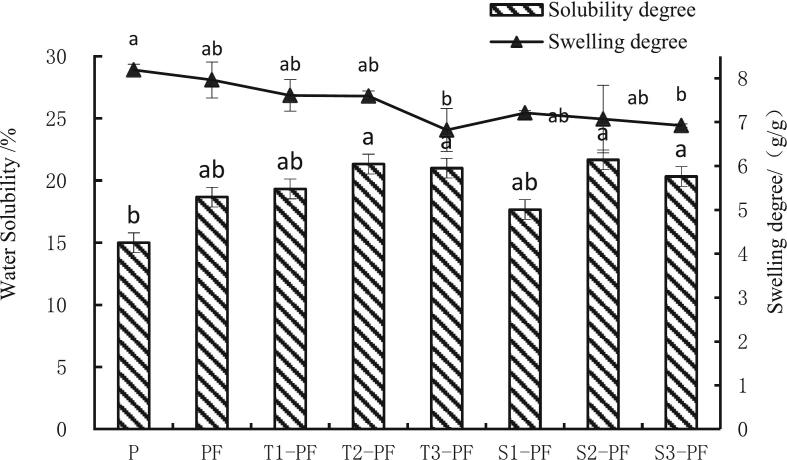
Solubility and swelling capacity of protein-fortified potato flour extruded at different temperatures and screw speeds. Note: All experiments were repeated at least thrice. The data are expressed as mean ± standard deviation, and different letters within a column are significant differences (*p* < 0.05).

The changes in solubility and swelling capacity of protein-fortified potato flour are shown in Fig. 7. The solubility reflects the degree of conversion of starch and the degradation of molecular structure during extrusion. The solubility of PF and extruded compound powder was higher than that of P and the solubility of S2-PF increased by 44.47 %. As the extrusion temperature and screw speed increased, the solubility increased, which occurred probably due to the degradation of starch during extrusion. The increase in the quantity of soluble polysaccharides released increased the solubility of the compound powder. Additionally, as the crosslinking reaction between starch and protein was better, more hydrophilic hydroxyl groups were introduced into the exogenous protein, and the protein molecules were degraded into fragments with low molecular weights that were more soluble in water under the action of high temperature and high shear(Chen et al., 2022); thus, the extruded compound powder had higher solubility. When the temperature and screw speed reached a certain value, the extrusion temperature and screw speed did not significantly affect the solubility of the sample (*p*>0.05).

The swelling capacity represents the water-holding capacity of starch after it disperses in a certain quantity of water. It also determines the stability of starch in water. The expansion force of PF was lower than that of P and the added exogenous protein molecules surrounded or adhered to the surface of starch granules to regulate the expansion of starch granules. The expansion forces of T1-PF, T2-PF, T3-PF, S1-PF, S2-PF and S3-PF were lower than those of P and PF, which implied that extrusion treatment enhanced the stability of the compound system. As the extrusion temperature and screw speed increased, the expansion force of the compound powder decreased. When the extrusion temperature and screw speed were higher, the expansion force was lower. This occurred probably because the starch polymer degraded in the extrusion, the structure was destroyed and its ability to absorb water decreased; these results were similar to those reported by Singh (Singh et al., 2015). Studies have shown that as the extrusion temperature increases, the degree of starch degradation and gelatinization increases, and the expansion force decreases.

### 3.8 Thermal properties

The effect of extrusion treatment on the thermal properties of compound potato powder is shown in Table 1, where the peak temperature (Tp) reflects the stability and anti-gelatinization ability of the starch granule structure. After extrusion treatment, the initial temperature (To) and peak temperature (Tp) of the compound potato powder increased significantly (*p* < 0.05). The Tp of samples expressed significant differences (*p* < 0.05) and the order was P < PF < T1-PF < T2-PF < S1-PF < S3-PF < T3-PF < S2-PF. This occurred due to the interaction between amylose and amylose and between amylose and amylopectin after extrusion treatment. The interplay of starch chains and reduced mobility of amylopectin elevated the thermal stability of the granules, thus increasing To and Tp. Additionally, the interplay between starch and protein during extrusion formed a more stable complex, which promoted the construction of the protein network structure, resulting in an increase in the temperature required to destroy the starch molecules, thus enhancing the thermodynamic stability of the compound powder. The results showed that samples in S2-PFgroup had the best thermal stability and gelatinization resistance. The gelatinization enthalpy (ΔH) is responsible for the heat energy required to destroy the double helix structure of starch during gelatinization, the starch with lower ΔH could gelatinize more easily (Wu et al., 2020). After extrusion treatment, the ΔH of T1-PF, T2-PF, T3-PF, S1-PF, S2-PF and S3-PF decreased with an increase in the temperature and screw speed. The results showed that the higher the extrusion temperature and screw speed, the lower the starch gelatinization energy. The ΔH of PF decreased from 1.71 J/g to 1.25 J/g. This occurred because, during the gelatinization of starch, the protein added to the complex hindered the interaction between starch and water, thus reducing ΔH. The ΔH of T3-PF and S3-PF is 43.86 % and 40.94 % lower than that of P, which occurred due to the destruction of the amorphous region after extrusion treatment. The double helix structure was reduced and only a small amount of heat was required for gelatinization.

**Table 1:** Thermal characteristic parameters by DSC.

Samples	To/°C	Tp/°C	Tc/°C	∆H/(J/g)
P	74.34 ± 0.44^d^	82.31 ± 0.24^d^	92.57 ± 0.45^a^	1.71 ± 0.03^a^
PF	76.17 ± 0.34^c^	83.01 ± 0.20^c^	89.97 ± 0.23^c^	1.25 ± 0.02^b^
T1-PF	76.89 ± 0.22^b^	83.45 ± 0.22^b^	90.33 ± 0.21^c^	1.21 ± 0.01^bc^
T2-PF	77.21 ± 0.18^b^	83.64 ± 0.33^b^	90.18 ± 0.14^c^	1.04 ± 0.06^d^
T3-PF	77.46 ± 0.27^b^	83.71 ± 0.04^b^	90.00 ± 0.17^c^	0.96 ± 0.00^f^
S1-PF	77.43 ± 0.71^b^	83.65 ± 0.15^b^	91.06 ± 0.15^b^	1.18 ± 0.02^c^
S2-PF	78.28 ± 0.16^a^	84.47 ± 0.43^a^	90.83 ± 0.11^b^	0.97 ± 0.01^ef^
S3-PF	77.37 ± 0.07^b^	83.70 ± 0.11^b^	89.94 ± 0.22^c^	1.01 ± 0.01^de^

Note: All experiments were repeated at least thrice. The data are expressed as mean ± standard deviation, and different letters within a column are significant differences (*p* < 0.05).

### 3.9 Thermal degradation

**Fig. 8:**
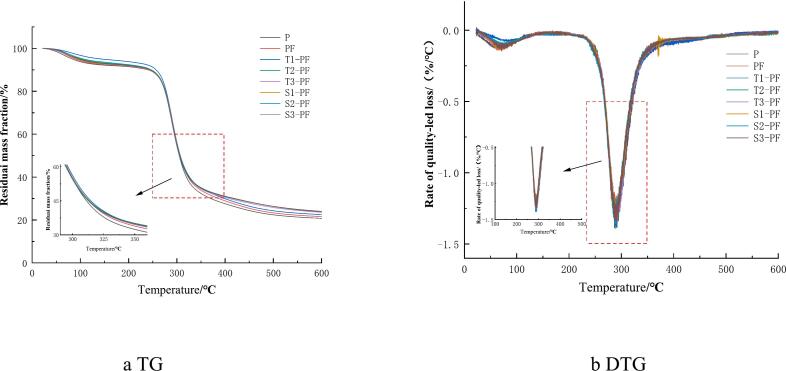
TG/DTG curves of protein-fortified potato flour extruded at different temperatures and screw speeds.

The TGA and DTG curves from thermogravimetric test of extruded protein-fortified potato flour are shown in Fig. 8. The depletion of mass in all the samples predominantly took place in three stages. The mass loss in the first stage occurred at 50–150 °C, which was mainly related to the evaporation of water in the sample. The mass loss in the second stage occurred at 150–350 °C, and the sample decomposed rapidly, mainly due to the thermal degradation of starch and protein molecules, mainly involving the cleavage of starch C—H, C—C, and hydroxyl groups and the breakage of covalent peptide bonds in proteins(Li et al., 2020). The maximum mass loss temperature of P was 327.43 °C and the maximum mass loss temperature of PF was 332.87 °C (Fig. 8). Compared to the changes recorded in P, the temperature corresponding to the maximum mass loss rate of PF at this stage increased and the mass loss rate of the compound powder decreased, indicating that adding protein improved the thermal stability of the compound potato powder. In this temperature range, T1-PF, T2-PF, T3-PF, S1-PF, S2-PF and S3-PF showed higher maximum mass loss rate temperatures than PF, and the maximum mass loss temperatures of the compound powder were 337.72 °C, 337.63 °C, 335.18 °C, 336.51 °C, 335.95 °C and 336.71 °C, respectively. Extrusion increased the heat dissipation temperature of the compound powder and reduced its mass loss rate, which occurred probably because of the production of protein-starch complexes during extrusion. The proteins were crosslinked with starch. During extrusion, the construction of the network structure of proteins was promoted. The promotion of the formation of the complex by extrusion treatment can improve the thermal stability of the complex, which was similar to the results reported by Chen (Chen et al., 2023). The thermal stability of the T3-PF sample was slightly lower than that of T1-PF and T2-PF, which may be related to the decrease in complex formation and the destruction of the structure caused by high-temperature extrusion treatment. The thermal degradation in the third stage occurred at 350–600 °C. At this stage, the curve was relatively flat and the sample was further thermally degraded or even completely decomposed.

### 3.10 Low-field nuclear magnetic resonance (LF-NMR)

**Fig. 9:**
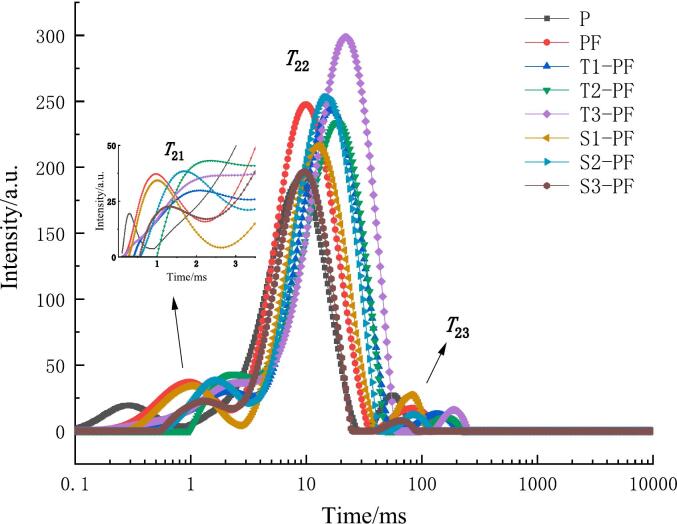
T_2_ inversion diagram of water transverse relaxation time of protein-fortified potato flour dough extruded at different temperatures and screw speeds.

The distribution of water in the dough was tested by low-field nuclear magnetic resonance to understand the migration of water between starch and protein and other components in the dough. Generally, T symbolizes the relaxation time for each water state and A symbolizes the ratio of the peak area of each state of water to the total area. The signal intensity of the transverse relaxation time map of different samples is shown in Fig. 9. Except for T3-PF, the other samples showed three characteristic peaks. T_21_ indicated deep-bound water tightly bound to starch and protein, T_22_ indicated weak-bound water and T_23_ indicated free water (Liu et al., 2019). The relaxation time (T_2_) of PF is higher than that of P (Fig. 9), indicating that the addition of foreign proteins results in increased water freedom and enhanced mobility. The T_2_ of T1-PF, T2-PF, T3-PF, S1-PF, S2-PF and S3-PF was significantly higher than that of P and PF (*p* < 0.05). This suggests that squeezing affects the degree to which water in the dough binds to other substances. When the starch particles are destroyed, the structure becomes less rigid and the ability of the starch particles to bind water molecules is weakened. With the increase of extrusion temperature, T_2_ increases. When the temperature reaches the maximum (T3-PF), only two peaks, T_22_ and T_23_, are found in the spectrum. High temperature affects the cross-linking of starch and protein, affects the formation of bound water, destroys the formation of protein network structure, and leads to the increase of water migration and relaxation time. Similarly, with the increase of screw speed, T_2_ of the sample first increased and then decreased, indicating that the water migration rate was higher at appropriate temperature and screw speed.

The content of water in different states is shown in Table 2. A_21_ A_22_ and A_23_ represent the peak area percentages of deep-bound water, weak-bound water and free water, respectively. The proportion of the three states of water in the dough followed the order A_22_ (weak bound water) > A_21_ (deep bound water) > A_23_ (free water). After extrusion treatment, the proportion of deep-bound water A_21_ in the compound powder dough was significantly higher than that in P (*p* < 0.05). An increase in the proportion of A_21_ increased the bound water content, improved the water-holding capacity and enhanced the structure stability of the dough. The free water in PF, T1-PF, T2-PF, T3-PF, S1-PF, S2-PF and S3-PF was significantly lower than that in P, which was related to the addition of exogenous protein. The exogenous proteins added formed crosslinking with starch and the protein components with a high water-binding capacity took up the water released from the starch matrix, thus reducing the free water in the system. Additionally, the protein in the dough formed a network structure, which increased the compactness of the dough structure, decreasing the loss of water molecules. As the extrusion temperature and screw speed increased, both the free water A_23_ and the fluidity in the extruded compound powder dough decreased. This indicated that the hydrophilic effect of the whole flour after extrusion treatment decreased the dough's free water content. As the temperature and screw speed increased, the ability of water molecules to be combined decreased, which matched the test results of water absorption capacity. This finding indicated that the gelatinized starch and modified protein after extrusion treatment affected the movement of water molecules and promoted the distribution of water in the dough.

**Table 2:** The transverse relaxation time and the corresponding peak area of protein-fortified potato flour dough extruded at different temperatures and screw speeds.

Samples	*T*_21_/ms	*T*_22_/ms	*T*_23_/ms	*A*_21_/%	*A*_22_/%	*A*_23_/%
P	0.29 ± 0.03^f^	8.74 ± 0.33^f^	55.08 ± 0.80^g^	7.6 ± 0.70^d^	88.1 ± 2.0^b^	4.3 ± 1.37^bc^
PF	0.96 ± 0.03^e^	9.97 ± 0.14^f^	83.89 ± 0.37^d^	12.4 ± 1.05^a^	84.3 ± 2.3^c^	3.2 ± 1.61^ab^
T1-PF	2.11 ± 0.10^b^	16.43 ± 0.32^c^	134.68 ± 3.07^c^	9.7 ± 0.79^bc^	88.2 ± 0.7^b^	2.1 ± 1.40^d^
T2-PF	2.35 ± 0.09^a^	18.74 ± 1.11^b^	161.90 ± 1.58^b^	10.3 ± 0.61^b^	88.4 ± 1.56^b^	1.3 ± 0.95^bc^
T3-PF		21.94 ± 1.19^a^	189.57 ± 1.06^a^		98.6 ± 0.5^a^	1.4 ± 0.50^bc^
S1-PF	1.01 ± 0.12^e^	12.63 ± 0.14^e^	79.59 ± 0.59^e^	12.1 ± 0.70^a^	84.1 ± 2.95^c^	3.8 ± 3.34^bc^
S2-PF	1.71 ± 0.01^c^	14.79 ± 0.81^d^	86.13 ± 0.55^d^	10.6 ± 0.70^b^	87.9 ± 0.5^b^	1.4 ± 0.92^bc^
S3-PF	1.32 ± 0.08^d^	9.71 ± 0.56^f^	64.49 ± 0.57^f^	8.5 ± 0.36^cd^	90.1 ± 0.66^b^	1.4 ± 0.70^bc^

Note: All experiments were repeated at least thrice. The data are expressed as mean ± standard deviation, and different letters within a column are significant differences (*p* < 0.05).

## 4 Conclusions

The twin-screw heat-moisture extrusion treatment altered the structure of starch granules and proteins, enhanced the interaction between starch and protein to form complexes, and increased the relative crystallinity of the compound powder, thereby promoting the development of a network structure in the compound powder dough, which increased the number of exposed binding sites, thereby improving WAC, OAC and solubility of the compound powder. Additionally, the strengthening effect of exogenous protein increased the competition of the original protein for water, inhibited the expansion and gelatinization of starch granules, and thus, improved the gelatinization enthalpy, the heat dissipation temperature and reduced its mass loss rate of compound powder. The thermal stability of the starch-protein complexes increased. After extrusion, the content of bound water in the dough increased. However, too high extrusion temperature and screw speed further destroyed the internal structure of starch and the network structure of proteins. The extrusion treatment at suitable temperature and speed effectively improved the structure and functional properties of protein-fortified potato flour. This study provides a theoretical framework for improving the processing performance of potato flour and promoting the application of potato flour in the field of gluten-free functional foods.

## Fund Project

10.13039/501100001809National Natural Science Foundation of China (32060582).

## CRediT authorship contribution statement

**Suying Hao:** Writing – original draft, Software, Methodology, Investigation, Data curation. **Ying Zheng:** Validation, Software. **Mingyuan Li:** Validation, Software. **Xiaobo Feng:** Validation, Software. **Xiaoqing Yang:** Writing – review & editing, Funding acquisition, Conceptualization.

## Declaration of competing interest

The authors declare that they have no known competing financial interests or personal relationships that could have appeared to influence the work reported in this paper.

## Data Availability

Data will be made available on request.
